# Comparative perianal fistula closure rates following autologous adipose tissue-derived stem cell transplantation or treatment with anti-tumor necrosis factor agents after seton placement in patients with Crohn’s disease: a retrospective observational study

**DOI:** 10.1186/s13287-021-02484-6

**Published:** 2021-07-13

**Authors:** Min Young Park, Yong Sik Yoon, Jong Lyul Lee, Sang Hyoung Park, Byong Duk Ye, Suk-Kyun Yang, Chang Sik Yu

**Affiliations:** 1 Department of Colon and Rectal Surgery, Asan Medical Center, University of College of Medicine, 88, Olympic-ro 43-gil, Songpa-gu, Seoul, 05505 Republic of Korea; 2 Department of Gastroenterology, Asan Medical Center, University of Ulsan College of Medicine, Seoul, Republic of Korea

**Keywords:** Adipose tissue-derived stem cell, ASC, Biologics, Crohn’s disease, Fistula

## Abstract

**Background:**

Perianal fistula is one of the most common complications in Crohn’s disease, and various medical and surgical treatments are being tried. The aim of this study was to compare the perianal fistula closure rates following treatment with anti-tumor necrosis factor (TNF) agents or autologous adipose tissue-derived stem cell (auto-ASC) transplantation with Crohn’s disease (CD).

**Methods:**

CD patients who underwent seton placement for perianal fistula from January 2015 to December 2019 at a tertiary referral center were retrospectively reviewed. Patients were divided into two groups, one that received sequential treatments with anti-TNF agents (anti-TNF group) and the other that underwent auto-ASC transplantation (stem cell group). Clinical variables and fistula closure rates were compared in the two groups.

**Results:**

Of the 69 patients analyzed, 39 were treated with anti-TNF agents and 30 underwent auto-ASC transplantation. Compared with the stem cell group, patients in the anti-TNF group were older (*p*=0.028), were more frequently male (*p*=0.019), had fistulas with more penetrating behavior (*p*=0.002), had undergone surgery more frequently (*p*=0.010), and had a shorter interval from seton placement to intended treatment (*p*<0.001). During a median follow-up of 46 months (range, 30–52.5 months), fistula closure rates were significantly faster (83.3% vs. 23.1%, *p*<0.001), and the mean interval from seton placement to fistula closure significantly shorter (14 vs. 37 months, *p*<0.001) in the stem cell than in the anti-TNF group. Three patients experienced fistula recurrence, all in the stem cell group.

**Conclusions:**

Medical treatment using anti-TNF agents and auto-ASC transplantation are feasible treatment options after seton placement for Crohn’s perianal fistula. However, the closure rate was significantly faster and the time to closure significantly shorter in patients who underwent auto-ASC transplantation than medical treatment.

**Trial registration:**

This study was retrospectively registered and approved by the Institutional Review Board of Asan Medical Center, number 2020-1059.

## background

Crohn’s disease is a chronic relapsing systemic inflammatory disease of unknown origin which interest the whole intestine, most frequently affecting the distal ileum [1, 2]. This transmural inflammation disrupts the integrity of the intestinal mucosa, favoring the development of abscesses and fistulas. When fistulas were formed, they can make tracks between intestines or between the intestine and other organs such as the bladder, vagina, adjacent tissue or the skin [3]. A perianal fistula is an abnormal communication between the rectum or anal canal and the external perianal or ischioanal skin [1]. Perianal fistula is one of the most common complications in Crohn’s disease (CD), with an estimated lifetime risk in western patients of 14% to 38% [4]. The incidence of perianal complications is higher in East Asian than in western patients, ranging from 30.3% to 58.8% [5]. Moreover, the incidence of CD is increasing in Asia, with 43% of CD patients in South Korea having perianal fistula [6].

Medical treatment for CD is mainly focused on the control of bowel inflammation, not fistula. Antibiotics are commonly used as a first-line therapy for fistula treatment, but have not been proven to be effective in treating Crohn’s perianal fistula (CPF) [7]. A meta-analysis of five studies found a response in 54% of patients treated with azathioprine or 6-mercaptopurine, but this meta-analysis was limited in that the literature reviewed was not a well-designed prospective clinical study and that response assessments to fistulas were made on different criteria with complete closure or reduced discharge as secondary endpoints [8]. Nowadays, biological agents such as anti-tumor necrosis factor (TNF)-α are increasingly being used to treat Crohn fistula [9, 10]. Because CD is associated with a T-cell mediated response, and the hallmark of pathogenesis is transmural inflammation, which is facilitated by increased proinflammatory cytokines, interferon-ɣ and interleukin-12, as well as TNF-α [11–14]. Previous studies reported that TNF-α is increased in the stool of patients with active CD compared to controls [15, 16]. Anti-TNF antibodies are thought to neutralize of TNF-α, reverse signaling, apoptosis, and cytotoxicity [17] and have a predilection and efficiency for distribution into inflamed tissue [18]. Anti-TNF therapies with an Fc region (infliximab and adalimumab) are also able to induce antibody-dependent cell mediated cytotoxicity and complement-dependent cytotoxicity [18]. In the ACCENT II trial, the closure rate of fistula was 63% at week 14 but decreased to 36% at week 54 [10]. Overall, the currently available treatments for CPF are not satisfactory as they do not achieve complete closure and reduction of recurrence. Although anti-TNF agents are an effective option for treatment of Crohn’s perianal fistula [19], treatment with biologic agents alone does not result in high cure rates [20, 21]. Rather, effective treatment of CPF requires a combination of biologic agents and surgery, with closure rates of 50–82% [22].

Given the problem of Crohn's fistula and the unmet medical needs, attention has been focused on stem cell therapy. Autologous or allogenic adipose tissue-derived stem cells (ASCs) may be safe and effective for the treatment of CPF. In a previous study, ASC transplantation resulted in closure of recto-vaginal fistula in patients with perianal Crohn's disease without any adverse events related to the treatment and recurrence [23, 24]. In addition, the other study reported higher quality of life scores and closure rates of ASCs than controls [25]. Autologous ASCs (auto-ASCs), which are commercially available in South Korea, have shown favorable result with 80.8% of 2-year closure rate [26]. In clinical practice, many patients with CPF initially undergo seton placement to control perianal inflammation and to maintain immunosuppressive medications such as azathioprine, mercaptopurine and methotrexate which reduce abnormal immune reactions. Multi-disciplinary teams (MDTs) can then decide on further treatment options, consisting of either biologic agents or surgery including auto-ASC transplantation.

Beyond conventional medical treatments such as immunosuppressive medications, the development of biologics that target specific mechanisms of the disease has resulted in more remission in Crohn’s disease patients [27]. Therefore, in Korea, where Crohn's disease is treated according to step-up approach, biologics are administered as the last step [28]. The use of biologics in South Korea has recently increased due to the expansion of medical treatment [29–31]. Moreover, the number of patients who have undergone perianal surgery during treatment with biologics has increased because of the high proportion of Korean CD patients with perianal fistulas [6, 29]. Currently, the types of biologics used in Korea include anti-TNF agents, anti-interleukin 12/23 agents and anti-α4β7 integrin agents. Among them, anti-TNF agents (infliximab and adalimumab) are mostly used. This study therefore compared outcomes in patients with CPF who underwent auto-ASC transplantation or were treated with anti-TNF agents.

## Methods

### Patients and clinical variables

Data were retrospectively reviewed in CPF patients who underwent seton placement from January 2015 to December 2019 at Asan Medical Center, Seoul, South Korea. CD was diagnosed by gastroenterologists based on clinical, endoscopic, radiological, and histopathologic criteria according to the diagnostic guidelines for CD in Korea [32]. There is no single gold standard for the diagnosis of CD and typical endoscopic findings of CD are non-continuous distribution of longitudinal ulcers, cobblestone mucosal appearance, and aphthous ulcerations arranged in a longitudinal fashion. The evaluation of small bowel with small bowel follow-through is recommended for suspected CD to establish diagnosis and to determine the extent and location of disease. Focal and patchy chronic inflammation, focal crypt irregularity, and non-caseating granulomas are usual microscopic features of CD. Patients who underwent examination under anesthesia, those without a detected fistula tract, patients with insufficient medical records, and those lost to follow-up were excluded. Patients were divided into an anti-TNF group and a stem cell group. The anti-TNF group included patients who received anti-TNF agent treatment for any purpose after seton drainage for perianal fistula. The stem cell group included patients who underwent auto-ASC transplantation after seton drainage for perianal fistula without receiving anti-TNF agents. The anti-TNF group therefore included patients who received at least one injection or infusion of anti-TNF agents within 3 months before or after surgery for treatment of perianal fistula, whereas the stem cell group excluded patients who received anti-TNF agents within 3 months before or after surgery for treatment of perianal fistula. Patients’ characteristics, including age, gender, smoking, and subclass of the Montreal classification [33], were compared. The Montreal classification describes the age of onset, extent and behavior of CD in more detail (Table 1). Fistula evaluation included fistula type (simple vs. complex, single vs. multiple), perioperative CD medication (immunomodulators or steroids) without anti-TNF agents, presence of proctitis or stricture, and presence of perianal abscess. Anti-TNF agents used in this study were infliximab (Remicade®, Janssen Biotech, Inc., Horsham, PA, USA) and adalimumab (Humira®, AbbVie, Inc., North Chicago, IL, USA). Auto-ASC used in this study was Cupistem® (Anterogen Co.,Ltd, Seoul, South Korea). The study protocol was approved by the Institutional Review Board of Asan Medical Center (No. 2020-1059).

**Table 1:** Montreal classification

Classification	Description
Age of onset (A)	
A1	16 years old or younger
A2	17-40 years old
A3	Over 40 years old
Location (L)	
L1	Terminal ileum
L2	Colon
L3	Ileocolon
L4	Upper gastrointestinal
Behavior (B)	
B1	Non-stricturing, non-penetrating
B2	Stricturing
B3	Penetrating

### Fistula types

**Fig. 1:**
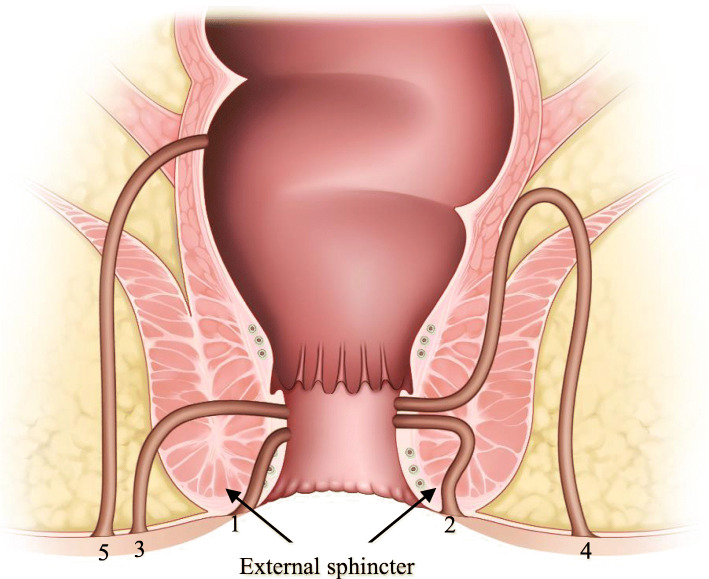
The Park’s classification of perianal fistulas. (1) Superficial, (2) Inter-sphincteric, (3) Trans-sphincteric, (4) Supra-sphincteric and (5) Extra-sphincteric

Fistulas were classified according to Park’s classification criteria [34]. Based on the Park’s classification, fistulas are categorized based on their position relative to the external sphincter (Figure 1). Fistulas were defined as intersphincteric when the tract penetrated the internal sphincter and coursed through the intersphincteric space to the perianal skin. Fistulas were defined as transsphincteric when the tract penetrated both the internal and external sphincters. Fistulas were defined as suprasphincteric when they crossed the internal sphincter and initially spread upwards to the intersphincteric space and then downwards, crossing the levator ani muscle before reaching the perianal skin. Fistulas were defined as extra-sphincteric when they originated from the rectal wall and coursed down through the levator ani muscle lateral to the external sphincter to reach the perianal skin, but did not penetrate the anal sphincter complex.

### Surgical procedures, anti-TNF agents, and postoperative management

**Fig. 2:**
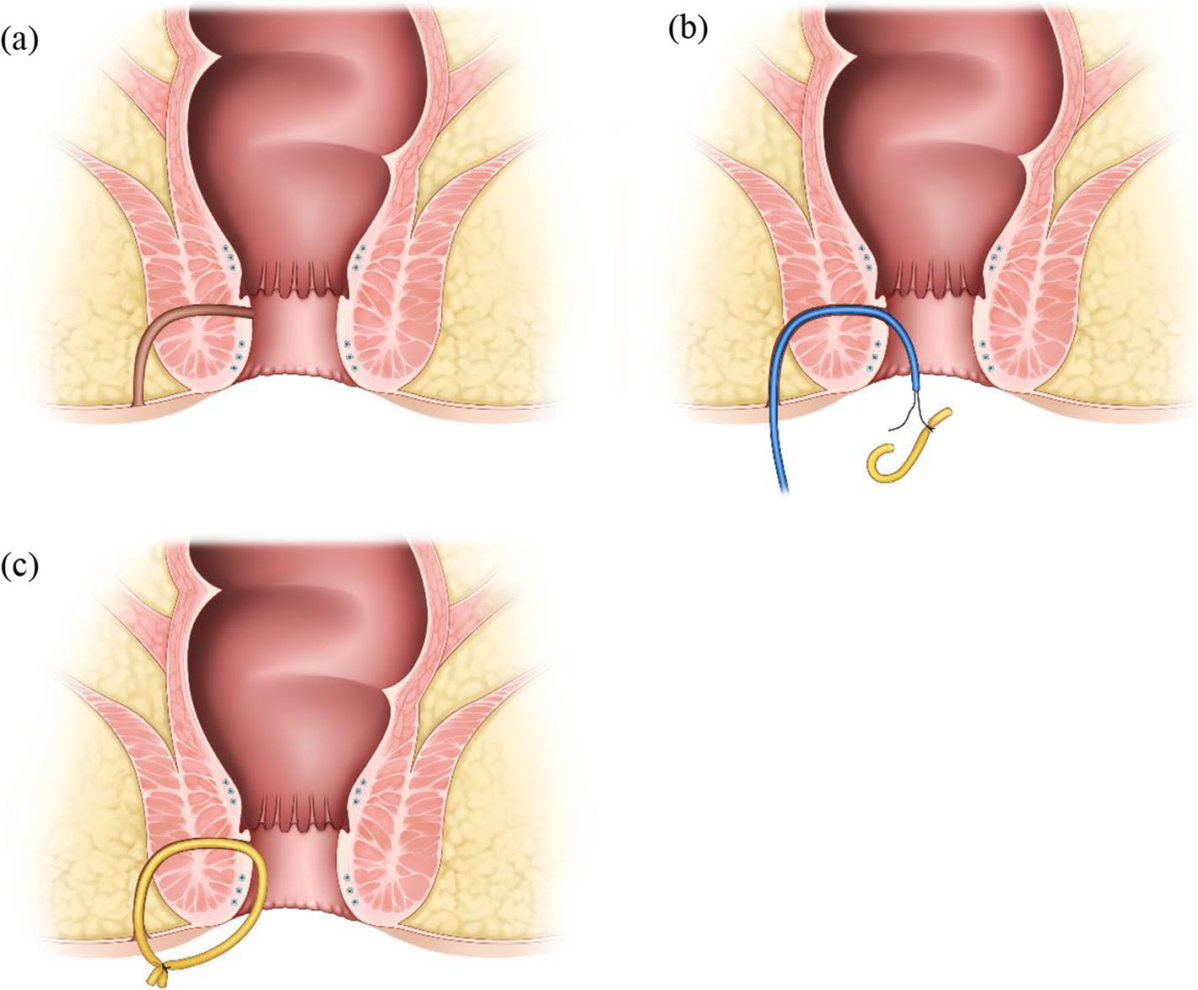
The procedures of seton placement. (**a**) Check the fistula tract. (**b**) Probe the fistula tract and apply a transsphincteric seton. (**c**) Tie the seton loosely as a noncutting seton drain

Seton placement: Under general anesthesia, the patient was placed in the prone jackknife position. The fistula tract was probed from the external openings and curetted, and the surrounding infected tissues were removed. A transsphincteric seton was applied and tied loosely as a noncutting seton drain (Figure 2). The type of seton drain was dependent on the type of fistula tract, with vessel loops used most frequently.

**Fig. 3:**
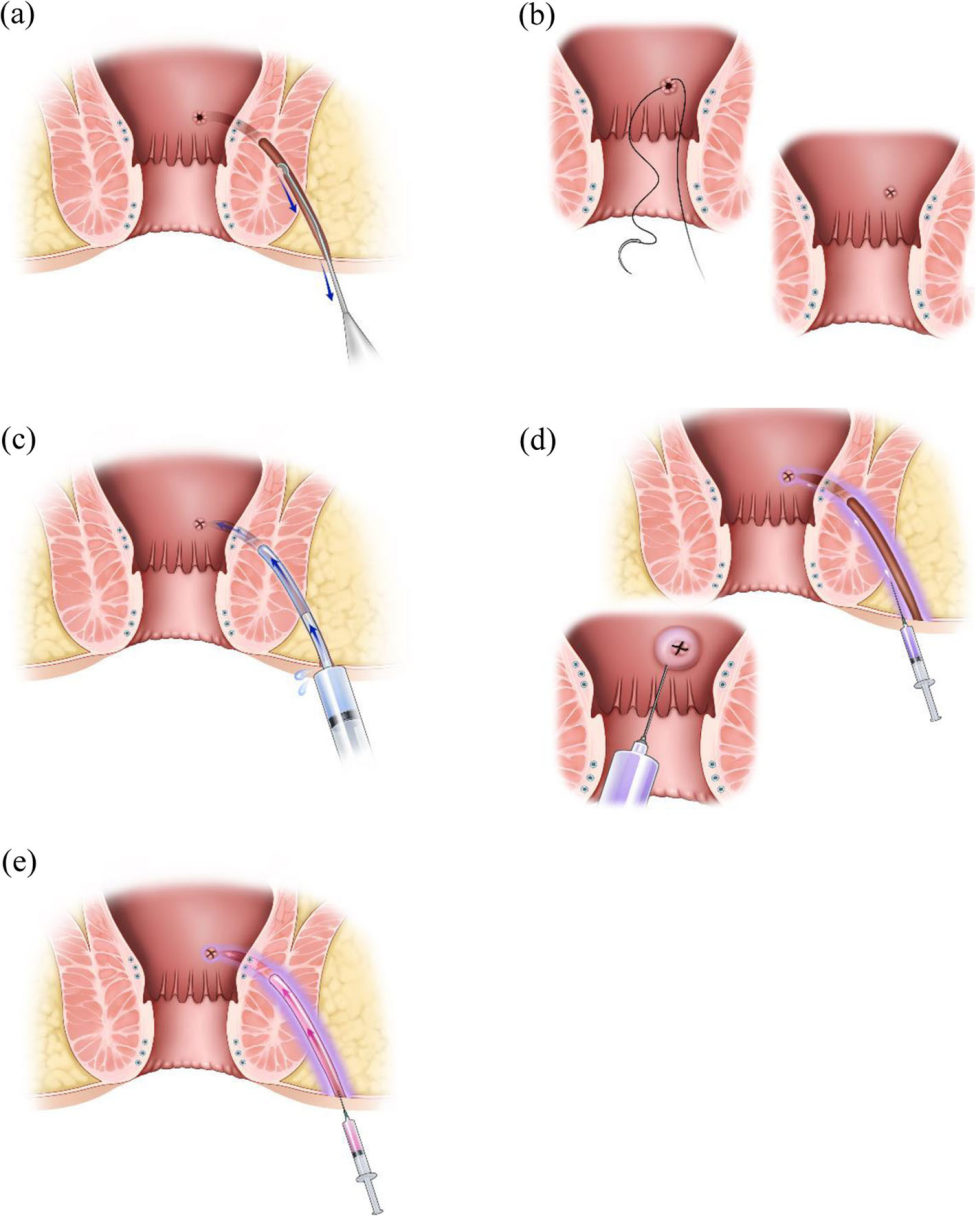
The procedures of auto-ASC transplantation. (**a**) Probe the fistula tract and curette the tract. (**b**) Close the internal opening of the tract. (**c**) Irrigate the tract with isotonic saline. (**d**) Inject stem cells into the submucosa around the internal opening and fistula tract

Auto-ASC transplantation: All candidates for auto-ASC transplantation underwent seton placement to control inflammation around the CPF. The tract was curetted to remove inflamed and fibrotic surrounding tissue, which prevents stem cell penetration. The tract was cleaned with isotonic saline, and the previous seton was removed. The internal opening of the tract on the rectal or anal canal was closed by direct suture ligation using vicryl. Stem cells were injected into the submucosa around the internal opening and fistula tract, and the opened fistula tract was filled with a mixture of stem cells and fibrin glue (Figure 3).

Anti-TNF agents: Infliximab was administered 2 and 6 weeks after the first dose, and 8 weeks after the third dose. Infliximab was administered 5mg/kg, and after that, when the reaction fell, the dose was increased to 10mg/kg. Adalimumab was administered every 2 weeks after the first dose. Adalimumab was administered at different doses in adults and children. In adults, the first 160 mg, 80 mg at 2weeks, 40mg at 4 weeks, and 40 mg every two weeks after that. In case of decreased response to adalimumab, it can be increased from every 2 weeks to every week. In children, it was different depending on the body weight. When the weight was less than 40kg, 40mg was initially administered, followed by 20mg ever two weeks. In the case of 40kg or more, 80mg was initially administered, followed by 40mg every two weeks [35, 36].

Postoperative management: Patients who underwent surgery were discharged home the next day and followed up at the outpatient clinic every 1 or 2 months. If there was no discharge or inflammation around the seton, seton removal was considered after discussion with the patient.

### Preparation of autologous ASCs

The auto-ASCs were transplanted with at a dose of 3×10^7^ cells/ml (Cupistem®, Anterogen Co., Ltd, Seoul, South Korea). These auto-ASCs were isolated from each patient’s subcutaneous fat tissue by lipo-aspiration. The lipo-aspirates were washed with phosphate buffered saline (PBS, Hyclone, Logan, UT, USA, http://www.hyclone.com) and digested in an equal volume of PBS containing 1% bovine serum albumin (BSA, Thermo Fisher Scientific, Penrose, AKL, NZ, https://www.thermofisher.com) and 0.025% collagenase type I (Thermo Fisher Scientific) for 80 minutes at 37°C with intermittent shaking. The stromal vascular fraction isolated from the fat tissue was cultured in Dulbecco’s modified Eagle’s medium (DMEM, Hyclone) with 10% fetal bovine serum (FBS, Hyclone) and 1 ng/ml human basic fibroblast growth factor (R&D, Minneapolice, MN, USA, http://www.rndsystems.com) to obtain the required number of ASCs for injection. The cells were harvested by trypsinization, suspended in DMEM, and packaged in single-use vials. All manufacturing procedures were carried out according to the Good Manufacturing Practices authorized by the Ministry of Food and Drug Safety (former KFDA). Prior to release, auto-ASCs were tested for cell appearance, viability, cell surface marker, in addition to adventitious agents including mycoplasma and bacteria, fungi, viruses and endotoxin.

### Outcomes

The primary outcome of the present study was a comparison of closure rates in the two groups. Closure of a fistula tract was defined as the absence of discharge, swelling, or pain. Recurrence was defined as a relapse of discharge and perianal symptoms, including pain and swelling, after closure of the fistulous tract without discharge.

### Statistics

Categorical variables were expressed as numbers and percentages and compared by chi-square tests. Continuous variables were expressed as medians and inter-quartile range (IQR) or mean ± standard deviation (SD) and compared by Student’s t-tests. Cumulative rates of fistula closure and perianal fistula recurrence were calculated using the Kaplan–Meier method and compared using log-rank tests. All statistical analyses were performed using SPSS for Windows, ver. 25.0 (SPSS Inc., Chicago, IL, USA), with p<0.05 considered statistically significant.

## Results

Of the 333 perianal surgeries for CPF, 239 (71.8%) involved seton placement; of the latter, 39 (16.3%) patients were treated with anti-TNF agents (anti-TNF group) and 30 (12.6%) underwent auto-ASC transplantation without anti-TNF agents (stem cell group). The 26 patients treated with anti-TNF agents and auto-ASC transplantation were excluded. Of the patients in the anti-TNF group, three were administered these agents mainly for CPF closure.

The clinical characteristics of the anti-TNF and stem cell groups are summarized in Table 2. Compared with the stem cell group, patients in the anti-TNF group were older (p=0.028), were more frequently male (p=0.019), and had fistulas with more penetrating behavior according to the Montreal classification (p=0.002). The mean albumin level tested before auto-ASC transplantation in the stem cell group or before seton placement in the anti-TNF group were statistically significantly higher in the stem cell group (3.36±0.48 vs. 4.05±0.42 g/dL, p<0.001), but were above 3.0 g/dL in both groups. In addition, the mean C-reactive protein (CRP) level tested at the same time were significantly higher in the anti-TNF group (3.03±3.04 vs. 0.56±0.74 mg/dL, p<0.001). The anti-TNF group had undergone surgery more frequently (p=0.010), and had a shorter interval from seton treatment to intended treatment (p<0.001).

**Table 2:** Demographic and clinical characteristics of patients in the anti-TNF and stem cell groups

	Anti-TNF (*n*=39)	Stem cell (*n*=30)	*p*-value
Age, median (IQR)	31.0 (25-34)	26.0 (20-30.25)	0.028
Sex			
Male	29 (74.4%)	14 (46.7%)	0.019
Female	10 (25.6%)	16 (53.3%)	
Montreal classification			
Age at onset			
A1 (≤16yr)	15 (38.5%)	6 (20.0%)	0.098
A2 (17-40yr)	24 (61.5%)	24 (80.0%)	
A3 (≥41yr)	0 (0.0%)	0 (0.0%)	
Location			
L1 (Ileum)	4 (10.3%)	8 (26.7%)	0.171
L2 (Colon)	3 (7.7%)	3 (10.0%)	
L3 (Ileocolon)	32 (82.1%)	19 (63.3%)	
Behavior			
B1 (Non-stricturing, non-penetrating)	12 (30.8%)	20 (66.7%)	0.002
B2 (Stricturing)	6 (15.4%)	6 (20.0%)	
B3 (Penetrating)	21 (53.8%)	4 (13.3%)	
Albumin, mean±SD (g/dL)	3.36±0.48	4.05±0.42	<0.001
CRP, mean±SD (mg/dL)	3.03±3.04	0.56±0.74	<0.001
Fistula type			
Simple	13 (33.3%)	6 (20.0%)	0.219
Complex	26 (66.7%)	24 (80.0%)	
Multiple fistula	15 (38.5%)	13 (43.3%)	0.683
Proctitis	19 (48.7%)	10 (33.3%)	0.199
Stricture	1 (2.6%)	2 (6.7%)	0.407
Abscess	18 (46.2%)	17 (56.7%)	0.387
Medical treatment			
Immunomodulators	16 (41.0%)	15 (50.0%)	0.458
Steroids	2 (5.2%)	0 (0.0%)	0.208
Smoking			
Current smoker	4 (10.3%)	1 (3.3%)	0.355
Ex-smoker	8 (20.5%)	4 (13.3%)	
Previous fistula OP	37 (94.9%)	30 (100%)	0.208
No. previous fistula OP, mean±SD (times)	3.24±1.89	2.17±1.29	0.010
Interval from seton treatment to anti-TNF or stem cell therapy, mean±SD (months)	1.03±0.93	16.13±16.01	<0.001
Disease duration, mean±SD (years)	9.37±6.13	6.10±4.88	0.018

Results are presented as n (%) unless otherwise indicated.

*IQR* Inter-quartile range, *TNF* tumor necrosis factor, *SD* Standard deviation, *CRP* C-reactive protein, *OP* operation

**Fig. 4:**
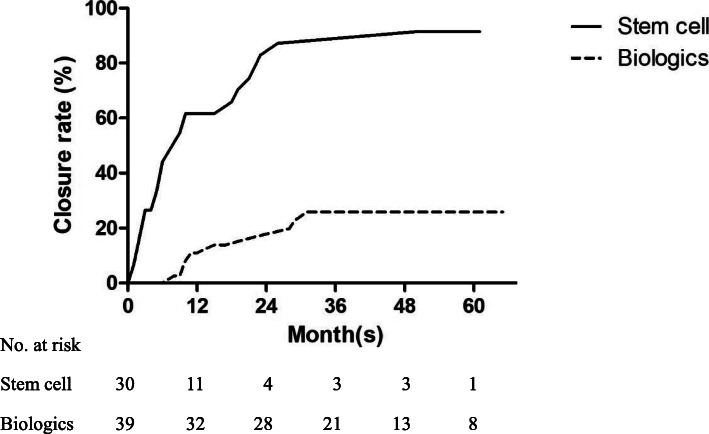
Kaplan–Meier analysis of fistula closure rates over time in auto-ASC transplantation and anti-TNF agents. Closure rate in patients who underwent seton treatment followed by auto-ASC transplantation without biologics is higher than in patients who underwent seton treatment followed by treatment with anti-TNF agents

During a median follow-up of 46 months (IQR, 30–52.5 months), closure rates were significantly higher in the stem cell group than in the anti-TNF group (83.3% vs. 23.1%, p<0.001; Figure 4). When adjusted the differences between the two groups, such as age, sex, behavior, albumin and CRP, closure rate was significantly faster in the stem cell group. Moreover, the mean time from seton placement to fistula closure was significantly shorter in the stem cell group than in the anti-TNF group (14 vs. 37 months, p<0.001). Three patients experienced fistula recurrence, all in the stem cell group. The recurrence risk showed no significant difference between the two groups. The mean time to recurrence was 21 months (Table 3). Recurrent abscesses in two of the patients were controlled by antibiotics, whereas the abscess in the third patient resolved spontaneously without any treatment.

**Table 3:** Rates of fistula closure and recurrence in patients in the anti-TNF and stem cell groups

	Anti-TNF (*n*=39)	Stem cell (*n*=30)	P by log-rank test	Unadjusted HR (95% CI)	*p*-value	Adjusted HR (95% CI)	*p*-value
Cumulative fistula closure, n (%)	9 (23.1%)	25 (83.3%)	<0.001	8.17 (3.72-17.94)	<0.001	3.15 (1.22-8.13)	0.018
1 year	4 (10.3%)	18 (60.0%)					
2 year	6 (15.4%)	23 (76.7%)					
3 year	9 (23.1%)	24 (80.0%)					
Closure time, mean±SD (months)	36.7±19.2	13.7±15.5			<.001		
Cumulative fistula recurrence, n (%)	0 (0%)	3 (10.0%)	0.288	2.64 (0.9-81.18)	0.578		
1 year	-	1 (33.3%)					
2 year	-	1 (33.3%)					
3 year	-	1 (33.3%)					
Recurrence time, mean±SD (months)	-	21.33±13.05	-				
Follow up, mean±SD (months)	42.9±16.0	39.2±18.6			0.385		

Results are presented as n (%) unless otherwise indicated.

*TNF* tumor necrosis factor, *SD* Standard deviation

## Discussion

Seton placement is the main type of perianal surgery used to control sepsis in patients with CPF. After CPF is controlled, it can be effectively treated with anti-TNF agents or auto-ASC transplantation. The present study compared these treatment modalities. Although auto-ASC transplantation is more invasive than medical treatment, it was found to be associated with a higher rate of and shorter time to fistula closure. Previous studies, however, have shown that combination treatment of biologics and surgery yielded excellent results [37, 38].

Medications, including antibiotics, immunosuppressants, and steroids, have shown limited efficacy in the treatment of CPF, with high recurrence rates after cessation of treatment [39–41]. Infliximab is a well-established agent for the treatment of CD and CPF [42], although anti-TNF antibody treatment has improved outcomes in patients with CD. A randomized trial showed that healing rates were significantly higher with infliximab (~36–55%) than with placebo (~13–19%) [10, 43]. The combination of medication and surgery was more effective than either alone, with complete healing rates of 52% and 43%, respectively [22]. Although anti-TNF therapy was combined with seton placement in our anti-TNF group, the CPF closure rate was lower than in previous studies. One of the possible reasons for the low closure rate in our anti-TNF group was uncoordinated treatment by gastroenterologists and colorectal surgeons. Most of our study population was enrolled before 2019, when an MDT approach, which includes gastroenterologists, colorectal surgeons, radiologists, and pathologists, was introduced in our institution for the treatment of inflammatory bowel disease. This MDT approach, involving combinations of surgery and medications, has been shown to be effective in the treatment of CPF. Another reason for the low closure rate in our anti-TNF group may be the absence of a strict protocol for seton removal, which is subjectively determined by individual surgeons. In addition, anti-TNF agents used in the present study were mainly used for control of bowel inflammation rather than fistula closure. Thus, patients showed more aggressive behavior such as penetration according to the Montreal classification and higher CRP level in the anti-TNF group than in the stem cell group.

Stem cell transplantation of auto-ASCs for CPF has been shown to be safe and effective. For example, a phase I trial in five patients with CD fistula resulted in 75% complete closure of the external opening [44]. A phase II study reported fistula closure in 56% of patients undergoing auto-ASC transplantation [25], and a phase III trial evaluating the efficacy of ASCs in complex fistulas resulted in fistula healing rates of approximately 40% at 6 months and 50% at 1 year [45]. Korean studies using auto-ASCs showed that the 1 and 2 year closure rates were 88% and 75% [26, 46], respectively, comparable to our results. Since 2014, auto-ASC transplantation for CPF has been supported by the Korean government, with insurance covering the cost of this procedure (Cupistem®, Antrogen Co., Ltd) for patients with refractory or recurrent fistulas not responding to conventional treatment for more than 3 months, and for patients with complex fistulas where sphincter damage is expected after conventional surgery.

Rates of CPF recurrence have been reported to vary, from 17.8% for low-type fistulas to 55.5% for high-type fistulas after seton placement [47]. Another study reported recurrence rates of 42.8% in patients who received infliximab or underwent seton drainage alone, and 18.2% in patients receiving both seton placement and infliximab treatment [20]. The 1 and 2 year recurrence rates in patients who underwent auto-ASC transplantation were reported to be 11% and 16% [26, 46], respectively, consistent with the 10% rate in the present study. Given the significant rate of recurrence of CPF in patients receiving infliximab or underwent seton placement, stem cell transplantation would be a efficacious and safe alternative for refractory CPF in CD [48].

This study had important limitations due to its retrospective design, including strong selection bias and weighted grouping, with the course of disease being more aggressive in the anti-TNF than in the stem cell group. In addition, the number of patients was small and events were infrequent, precluding propensity score matching, multivariate analysis or adjustment. Actual fistula healing rate after treatment was not evaluated by imaging modalities, such as magnetic resonance imaging. The low rate of closure in the anti-TNF group may have been affected by uncoordinated treatment before the institution of an MDT approach. Further studies in larger numbers of patients treated according to a defined MDT approach are needed to evaluate the actual effects of both treatment modalities.

## Conclusion

In conclusion, medical treatment using anti-TNF agents and auto-ASC transplantation are feasible treatment options after seton placement for CPF. However, auto-ASC transplantation showed a higher closure rate than anti-TNF treatment in the present study.

## Data Availability

The datasets used and/or analysed during the current study are available from the corresponding author on reasonable request.

## Abbreviations

Anti-TNF: Anti-tumor necrosis factor
CD: Crohn’s disease
CPF: Crohn’s perianal fistula
ASC: Adipose tissue-derived stem cell
MDT: Multi-disciplinary team
PBS: Phosphate buffered saline
DMEM: Dulbecco’s modified Eagle’s medium
FBS: Fetal bovine serum
IQR: Inter-quartile range
